# Ontogeny of Adaptive Antibody Response to a Model Antigen in Captive Altricial Zebra Finches

**DOI:** 10.1371/journal.pone.0047294

**Published:** 2012-10-09

**Authors:** Tess L. Killpack, William H. Karasov

**Affiliations:** 1 Department of Zoology, University of Wisconsin, Madison, Wisconsin, United States of America; 2 Department of Forest and Wildlife Ecology, University of Wisconsin, Madison, Wisconsin, United States of America; CNRS, Université de Bourgogne, France

## Abstract

Based on studies from the poultry literature, all birds are hypothesized to require at least 4 weeks to develop circulating mature B-cell lineages that express functionally different immunoglobulin specificities. However, many altricial passerines fledge at adult size less than four weeks after the start of embryonic development, and therefore may experience a period of susceptibility during the nestling and post-fledging periods. We present the first study, to our knowledge, to detail the age-related changes in adaptive antibody response in an altricial passerine. Using repeated vaccinations with non-infectious keyhole limpet hemocyanin (KLH) antigen, we studied the ontogeny of specific adaptive immune response in altricial zebra finches *Taeniopygia guttata*. Nestling zebra finches were first injected at 7 days (7d), 14 days (14d), or 21 days post-hatch (21d) with KLH-adjuvant emulsions, and boosted 7 days later. Adults were vaccinated in the same manner. Induced KLH-specific IgY antibodies were measured using ELISA. Comparisons within age groups revealed no significant increase in KLH-specific antibody levels between vaccination and boost in 7d birds, yet significant increases between vaccination and boost were observed in 14d, 21d, and adult groups. There was no significant difference among age groups in KLH antibody response to priming vaccination, yet KLH antibody response post-boost significantly increased with age among groups. Post-boost antibody response in all nestling age groups was significantly lower than in adults, indicating that mature adult secondary antibody response level was not achieved in zebra finches prior to fledging (21 days post-hatch in zebra finches). Findings from this study contribute fundamental knowledge to the fields of developmental immunology and ecological immunology and strengthen the utility of zebra finches as a model organism for future studies of immune ontogeny.

## Introduction

The adaptive humoral (or ‘antibody-mediated’) immune system of birds comprises a highly protective, specific arm of defense that retains long-term memory of invading pathogens [1], [2]. The model for avian humoral immune function proposes that production of adequate numbers of peripheral B-cell lineages that express functionally different immunoglobulin (Ig) specificities requires at least four to six weeks to develop in all birds [3]. This model is derived from studies of precocial galliformes, in which B-cell development begins embryonically during approximately the final week of egg incubation and is completed approximately three to six weeks after hatching, well before chickens reach adult size [2]–[4]. However, some altricial bird species, which have short incubation times and among the fastest growth rates of land animals [5], [6], fledge at adult size less than four weeks after the start of embryonic development. Given the proposed model of humoral immune development, and evidence that maternal antibodies (Igs) from the egg are catabolized within two weeks post-hatch in altricial birds [7]–[9], it is likely that rapidly-developing altricial birds are immunologically immature and relatively vulnerable to infection throughout the nestling and early post-fledging periods.

Non-infectious antigen vaccinations and antigen-specific immune assays have been used to examine induced antibody responses in developing birds. Upon first exposure to an antigen, a primary antibody response predominated by IgM is generated, isotype class-switching occurs, and memory B-cells are stored. Upon repeated exposure to the same antigen, a rapid secondary, memory response occurs, predominantly in the form of IgY antibodies [1], [2]. Capacity for specific primary antibody response was detectable in altricial pigeons, pied flycatchers, and American kestrels injected with foreign antigen during the first week post-hatch [10]–[12]. Primary response levels were been shown to increase and become more reliable after the first week post-hatch in altricial pigeons and macaws [10], [13], which was in agreement with studies of response levels in precocial chickens and turkeys [14]–[16].

Fewer studies have examined the ontogeny of secondary, adaptive antibody responses in altricial birds. Pigeons initially injected with sheep red blood cells (SRBC) during the first 10 days post-hatch produced IgY antibodies when a second immunization was administered 2–3 weeks later, indicating that the capacity for class-switching from IgM to IgY and memory cell formation occurs within the first weeks of hatching. Secondary antibody responses to repeated injections of SRBC and the non-pathogenic conjugate dinitrophenol keyhole limpet hemocyanin (DNP-KLH) were studied in American kestrels using hemagglutination and ELISA assays, respectively [17]. Kestrels first injected at 10 days post-hatch and boosted with the same antigen 6 days later showed robust secondary antibody responses, though detection of responses to DNP-KLH using ELISA was more sensitive than the SRBC hemagglutination assay [17]. More detailed studies of age-related changes in adaptive secondary antibody response of kestrels found significantly higher secondary responses in birds first vaccinated with DNP-KLH at 7–9 days post-hatch compared with those vaccinated at 3–5 days post hatch, and that adult secondary response was four times higher than that of nestlings [12]. Based on these data, it is likely that altricial birds continue to develop adaptive immune function throughout the nestling period, though it is unclear whether nestlings and recent fledglings experience a period of susceptibility to infection before mature adaptive IgY levels are reached.

We present the first study, to our knowledge, to detail the ontogeny of specific adaptive antibody response in an altricial passerine, the zebra finch *Taeniopygia guttata*, throughout the entire nestling period. Studies of age-related changes in antibody response in zebra finches will contribute knowledge to the fields of comparative immunology and ecological immunology. Additionally, the elaboration of zebra finches as a model is of interest given that their genome has been sequenced and organismal-level studies could provide fundamental knowledge for integrative studies of immune function at a variety of biological levels, including the molecular level. This work aims to address two research questions: i) when are zebra finches capable of mounting a detectable specific secondary antibody response post-hatch? and, ii) does magnitude of secondary adaptive antibody response reach adult levels prior to fledging (21 days post-hatch) in zebra finches? We vaccinated zebra finch nestlings and adults with keyhole limpet hemocyanin (KLH) and detected adaptive KLH-specific antibody response using ELISA. KLH is a model antigen in immunology studies and is a T-cell dependent antigen, providing insight into the integrated ontogeny of both B- and T-cell mediated responses in zebra finches [12], [18]. We used guidelines from previous studies of immunological development in altricial birds [12], [17] as well as a preliminary study using adult male zebra finches to ensure effective stimulation of the immune system of developing zebra finch nestlings and detection of an antibody response, if present.

## Methods

### Ethics Statement

All experimental procedures were approved by the University of Wisconsin, Madison Animal Care and Use Committee (permit no. 1370).

### Birds

A breeding colony of adult zebra finches was maintained under standardized conditions of 14 h: 10 h L:D photoperiod (lights on 0600 h), 21–24°C temperature, and 40–50% relative humidity. Birds were provided with a seed diet consisting of commercial seeds and Mazuri Small Bird Breeder food supplement, cuttlefish bone (calcium source), grit, and water *ad libitum*. Enrichment foods of green vegetables and egg food mixture (hard-boiled eggs with shells, dried Volkman Featherglow egg food, and vitamin and mineral supplement) were provided to birds three times per week. Breeding pairs of adult zebra finches were taken from our stock population and housed in individual breeding cages. Pairs were provided with nest boxes and nesting material at the time of pairing. Nests were monitored daily after the onset of egg-laying to determine the exact hatch date of each nestling.

### Vaccine Preparation

We conducted a preliminary study of adult male zebra finches to determine a combination of antigen (Keyhole limpet hemocyanin (KLH) or Bovine serum albumen), adjuvant (Complete Freund’s Adjuvant (CFA), Montanide ISA720, or Titermax), and sampling time (7 or 9 days post-injection) that ensured effective stimulation of the immune system of zebra finch nestlings and detection of an antibody response, if present. Given the magnitude of the effect and variance, we determined that we had the most power to generate and detect responses using the combination of KLH antigen with CFA adjuvant (or Titermax adjuvant for boost), and that 7 days post-injection was a sufficient sampling time. Therefore, for the ontogeny study, vaccine formulations were prepared by diluting KLH antigen (Calbiochem #374817) in phosphate buffered saline at a dose of 2.0 µg antigen per g bird body mass. Diluted antigen was emulsified with Complete Freund’s Adjuvant (or Titermax adjuvant for boost) at a ratio of 50∶50 diluted antigen to adjuvant, per manufacturer instructions. Vaccine emulsions were prepared using the vortex method and verified using the ‘drop test’ [19].

### Antibody Production in Nestlings and Adults

Upon hatching, zebra finch nestlings were assigned to one of three groups for initial sampling and vaccination: 7 days (7d), 14 days (14d), or 21 days (21d) post-hatch (n = 13 for 7d and 14d groups, n = 14 for 21d group). A baseline blood sample (≤70 µL) was taken from zebra finch nestlings and previously unvaccinated adults (n = 14) for measurement of background absorbance signal on the KLH-specific ELISA. Birds were then injected in the pectoralis muscle with 50 µL KLH-CFA emulsion prepared at a dose of 2.0 µg antigen per g bird body mass. Nestlings at 7 days post-hatch, too small for baseline blood sampling by veinipuncture and subsequent vaccination, were euthanized and decapitated for collection of a baseline blood sample, and sibling 7-day nestlings were injected intramuscularly in the pectoralis with 25 µL KLH-CFA emulsion (2.0 µg antigen per g bird body mass). A blood sample was taken 7 days post-priming vaccination from injected birds in all age groups by puncturing the brachial vein with a 28-gauge needle and collecting the blood into heparinized microcapillary tubes (≤70 µL per bird). Nestlings and adults were then immediately boosted with 50 µL of the same dose of KLH (2.0 µg antigen per g bird body mass) emulsified with Titermax (1 week post-priming vaccination), and a post-boost sample was taken 7 days later (≤70 µL per bird). Blood samples were centrifuged and plasma was stored at −20°C until analysis of antibodies. Body mass and hematocrit were measured at the time of blood sampling. At the time of KLH injections, all birds were also injected with a commercial West Nile Virus (WNV) vaccine, for the purposes of another study. We have no reason *a priori* to expect the WNV vaccine to interfere with measurement of KLH-specific antibody response in our ontogeny study. A pilot study comparison of adult zebra finches simultaneously injected with KLH and WNV (n = 9) compared with those injected with KLH alone (n = 11) showed no significant difference in post-vaccination (p = 0.77) or post-boost (p = 0.50) KLH antibody response (data not shown). If there were an effect of WNV on KLH-specific antibody response in our ontogeny study, we anticipate that it would be uniform across treatments, given that all birds injected with KLH were also always injected with WNV.

### Specific IgY Antibody Detection using Enzyme-linked Immunosorbent Assay (ELISA)

Adaptive immune function was measured using an ELISA for KLH-specific IgY antibodies. Flat-bottomed 96-well plates were coated with 50 µl/well of KLH antigen (Calbiochem #374807) diluted to 0.5 mg/ml of KLH antigen in carbonate-bicarbonate coating buffer. Plates were incubated overnight at 4°C. Plates were washed four times with wash buffer (PBS+Tween) to remove unbound coating antigen, blocked with 100 µL/well of blocking buffer (5% nonfat milk+wash buffer) and incubated for 1 hour at 37°C. Blocking buffer was discarded and 100 µl of zebra finch plasma samples (and positive and negative plate control samples), diluted 1∶100 in blocking buffer, were added to the wells in duplicate and the plates were incubated for 1 hour at 37°C. Plates were washed four times with wash buffer, 50 µl of horseradish peroxidase-conjugated goat-anti-bird IgG ( = IgY) antibody (Bethyl Laboratories, Inc. #A140-110P) diluted 1∶700 in wash buffer was added to each well, and the plates were incubated for 1 hour at 37°C. Plates were washed four times with wash buffer, 100 µl of ABTS substrate was added to all wells, and the plates were incubated in the dark at room temperature for 10 minutes. Optical densities of the wells were read at 405 nm using an automated plate reader and average absorbance values of duplicate wells were recorded. All samples from a given bird were run on the same plate and samples from birds of different treatment groups were included on each plate. Average absorbance values for post-priming and post-boost samples were corrected for background absorbance values on KLH-specific ELISA by subtracting the average absorbance value of the pre-injection blood sample for each bird (or from a sibling bird, in the case of the 7d group).

### Statistics

Results are given as means ± sem (N = number of individuals). All tests were carried out using R statistical package [20]. Assumptions of normality and homoscedasticity were examined prior to use of parametric tests and log-transformation (after adding a constant to the corrected antibody response values to make them positive and non-zero) was performed to meet normality assumptions. ANOVA was used to compare absorbance values in pre-vaccination samples, body mass, and hematocrit among age groups. Linear mixed models of repeated measures of specific antibody response were fit using maximum likelihood criteria (including individual bird and parental breeding pair as random effects), and chi-square-based tests were used to determine the significance of fixed and random effects. Non-significant variables were iteratively removed from the model. Post-hoc Tukey’s contrasts distinguished differences among groups. In all tests, the significance level was set at p<0.05.

## Results

Baseline absorbance values from pre-vaccination samples, which likely measure cross-reactivity of ELISA reagents with non-KLH specific antibodies in samples, significantly differed among age groups (F_3,44_∶ 6.5, p<0.001). Adults (0.179±0.026) had significantly higher baseline absorbance values than all of the nestling groups. Nestlings at 7d (0.106±0.007), 14d (0.111±0.016), and 21d (0.096±0.006) post-hatch did not significantly differ from each other in baseline absorbance values. To correct for differences among age groups, baseline absorbance values were subtracted from post-priming vaccination and post-boost antibody response values for each bird (or sibling bird in the case of the 7d group).

**Figure 1:**
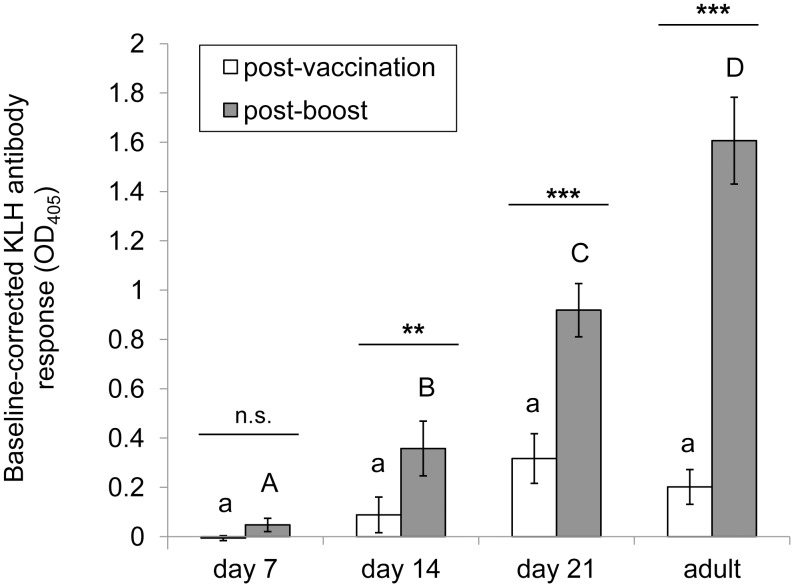
KLH-specific antibody response in vaccinated zebra finch nestlings and adults. Data are means ± s.e.m. (n = 13 for 7d and 14d groups, n = 14 for 21d and adult groups). Post-priming vaccination (unfilled bars) and post-boost (filled bars) absorbance values were corrected for absorbance values of baseline blood samples on KLH-specific ELISA. Post-vaccination values among age groups that share the same lowercase letter and post-boost values among age groups that share the same uppercase letter are not significantly different as determined by Tukey’s *post hoc* tests. Significant differences between post-vaccination and post-boost values within an age group (as determined by Tukey’s *post hoc* tests) are denoted by asterisks.

Repeated measures analysis of corrected KLH antibody response revealed a significant interaction of age and sampling time (Chi-square: 53.94, d.f. 3, p<0.001) (Figure 1). Comparisons within an age group revealed no significant increase in KLH-specific antibody levels between vaccination and boost in 7d birds (p = 0.992), yet highly significant increases between vaccination and boost were observed in birds vaccinated at 14d (p = 0.005) and 21d (p<0.001) and as adults (p<0.001).

There was no significant difference among age groups in corrected KLH antibody levels in response to priming vaccination (Figure 1), yet there were significant differences in corrected KLH antibody levels post-boost, with responses at 7d<14d<21d< adults (Figure 1). Post-boost antibody responses in all nestling age groups were significantly lower than in adults, indicating that mature adult secondary antibody response level was not achieved in zebra finches prior to fledging (Figure 1).

Hematocrit (Chi-square <0.001, d.f. 1, p>0.99) and body mass (Chi-square: <0.001, d.f. 1, p: 0.994) were not significant factors in predicting KLH-specific antibody response. Parental breeding pair was a significant random effect for corrected antibody response (Chi-square: 5.42, d.f. 1, p: 0.020). Vaccination did not significantly impact body mass or hematocrit in growing birds, as birds in different vaccination groups showed no significant differences in body mass or hematocrit measures at a given blood sampling age (data not shown).

## Discussion

We present the first study, to our knowledge, to detail the age-related changes in adaptive antibody response in an altricial passerine. Adaptive antibody response was not detectable in birds first injected at 7 days post-hatch, as there was a lack of significant increase in KLH-specific IgY between priming injection and boost. Significantly elevated levels of KLH IgY were detected post-boost in 14d, 21d and adult age groups, indicating capacity for adaptive antibody response. Lack of significant increase in 7d birds under our sampling regimen may be a result of an incompletely developed cell system, resulting in a more prolonged process of Ig class-switching in younger birds. Turkey poults injected with *Brucella abortus* antigen on the day of hatch reached peak antibody response at 15 days post-injection (dpi), whereas the specific antibody response of birds injected at 3 weeks post-hatch peaked sooner at 7dpi [15], indicating that the number and the functional competence of the B-cells likely increased with age. Detailed study of lymphocyte subpopulations and of the kinetics of antibody response over a range of times post-injection would shed light on the time course of Ig class switching and of the response of specific memory B-cell clones in altricial birds at different ages post-hatch.

In agreement with a previous study in American kestrels, we found that magnitude of adaptive antibody response gradually increases with age, yet is significantly lower than mature adult levels throughout the nestling period. We found that adaptive antibody response in zebra finches did not reach adult levels prior to fledging (21 days post-hatch in zebra finches), when free-living birds leave the nest and experience a more diverse antigenic environment.

Given that nestling birds have limited adaptive immune defenses, they may rely on other immune defenses for resistance. The lack of mature functional antibody response may be partly compensated by maternal provisioning of IgY via the egg yolk, which is absorbed into nestling circulation during development. However, studies of altricial passerine nestlings have shown that maternally derived antibodies are catabolized from circulation within two weeks post-hatch [7]–[9], which, according to our study, overlaps with the time that nestlings have immature endogenous humoral immune response capacity. Lack of mature adaptive immune function in growing nestlings may be compensated by endogenous innate immune defenses because their development does not require generation of diverse receptor specificities or memory cell lines [21], [22]. However, a recent study of tree swallows throughout the nestling period demonstrated that the bactericidal ability of whole blood was at sub-adult level at fledging age [23]. Levels of other innate immune measures, such as natural antibodies and complement, have also been shown to be at immature levels near fledging age in altricial passerines [24], [25]. Alternatively, nestlings may rely more on tolerance of infection while immune system components develop. Given that birds early post-hatch are overwhelmed with large quantities of novel foreign antigen and have high energetic demands of growth and development, it may be energetically cheaper to tolerate infection and limit damage rather than invest in immune defenses [26]. Given these previous studies of altricial passerines, this study provides another instance of immunological immaturity and is consistent with the hypothesis that passerine birds are particularly susceptible throughout the nestling period and immediately after fledging. More integrated studies of age-related changes in both passive and active components of immune resistance as well as tolerance will continue to shed light on the development of effective immune defense in growing altricial birds.

Knowledge of the ontogeny of adaptive immune defense in altricial passerines could have important implications for disease ecology. Passerines play an important role in the spread of zoonotic diseases in the wild, and nestling passerines in particular may be important hosts for vector-borne diseases given their undeveloped defenses and confinement to nests in close proximity to others, making them vulnerable to virus-carrying insects [27], [28]. Because detection of active viral infections can be difficult [28], wild birds are often caught and screened for virus-specific antibodies to provide evidence of a past infection. Knowledge of the ontogeny of antibody response can contribute to understanding of patterns of natural viremia in free-living nestlings and to effectively evaluate nestling exposure to zoonotic diseases.

A number of studies have examined the link between body mass and immune response in young birds, to examine proposed trade-offs associated with growth and immune processes competing for shared resources [29]. Adaptive humoral defenses are proposed to incur low costs of use because they are associated with anti-inflammatory cytokines [1], [30]. However, development of the adaptive humoral components may be relatively costly, given that generation and rapid proliferation of B-cell lineages with diverse, functional receptor specificities is confined to the energetically demanding developmental period of most vertebrates [30]. Reduced body mass growth was observed in young antigen-challenged precocial quail [31] yet vaccination did not interfere with growth in challenged altricial American kestrels [12] or the zebra finches in our study. Because the developing birds in our study were provided with *ad libitum* food, it is difficult to evaluate the relative costs of immunity. A study of skin-swelling response to phytohemagglutinin (PHA) in captive sand martins (*Riparia riparia)* demonstrated that the magnitude of PHA response was positively correlated with body mass growth when animals were fed *ad libitum* and negatively correlated with body mass growth when food was restricted 40% for three days [32], highlighting the importance of considering energy intake when evaluating trade-offs in growth and immune response. Future studies of the ontogeny of immune function in birds should examine the relationship between growth and antibody response in the context of a variety of ecologically relevant feeding scenarios to examine the biological costs of developing and mounting specific antibody responses and the sustained impact of environmental perturbations on immune defense and fitness in free-living animals.

Lastly, we found that parental breeding pair was a significant predictor of antibody response, perhaps indicating a genetic component to the magnitude of antibody response. Genetic-based constraints have been posited to influence the strength of immune response [33] and likely contribute to variability in antibody response to diverse antigens in growing birds [14], [16], [33]. Future experiments should include use of cross-fostering designs or examination of genes involved in recognition (including highly polymorphic MHC loci) to more deliberately examine the contribution of genetic constraints to variability in immune response during development in altricial birds. Additionally, comparative studies of diverse antigens (e.g. live infection versus inert antigens, T-cell dependent versus independent antigens) could contribute knowledge to variability in immune ontogeny in growing birds.

### Conclusions

We found that altricial zebra finch nestlings hatch with limited capacity for endogenous adaptive antibody defense and that capacity gradually increases throughout the nestling period. We found that magnitude of adaptive antibody response of all nestling age groups was significantly lower than that of adults, indicating that completion of immunological development occurs post-fledging in zebra finches. This study serves as a framework for future studies investigating development of immune resistance and tolerance, as well as physiological and fitness consequences of antigen exposure and infection during the energetically demanding nestling period of altricial birds.
